# Early risk factors and the role of fluid administration in developing acute respiratory distress syndrome in septic patients

**DOI:** 10.1186/s13613-017-0233-1

**Published:** 2017-01-23

**Authors:** Raghu R. Seethala, Peter C. Hou, Imoigele P. Aisiku, Gyorgy Frendl, Pauline K. Park, Mark E. Mikkelsen, Steven Y. Chang, Ognjen Gajic, Jonathan Sevransky

**Affiliations:** 1 Division of Emergency Critical Care Medicine, Department of Emergency Medicine, Brigham and Women’s Hospital, 75 Francis St., Neville House, Boston, MA 02115 USA; 2 Surgical ICU Translational Research (STAR) Center, Brigham and Women’s Hospital, Boston, MA USA; 3 Department of Anesthesiology, Perioperative and Pain Medicine, Brigham and Women’s Hospital, Boston, MA USA; 4 Division of Acute Care Surgery, Department of Surgery, University of Michigan Health System, Ann Arbor, MI USA; 5 Division of Pulmonary, Allergy, and Critical Care Medicine, Department of Medicine, Perelman School of Medicine, University of Pennsylvania, Philadelphia, PA USA; 6 Division of Pulmonary and Critical Care, Department of Medicine, UCLA, Los Angeles, CA USA; 7 Division of Pulmonary and Critical Care Medicine, Department of Medicine, Mayo Clinic, Rochester, MN USA; 8 Division of Pulmonary, Allergy and Critical Care, Department of Medicine, Emory University, Atlanta, GA USA

**Keywords:** Sepsis, Acute respiratory distress syndrome, Fluid resuscitation, Pneumonia, Acute lung injury

## Abstract

**Background:**

Sepsis is a major risk factor for acute respiratory distress syndrome (ARDS). However, there remains a paucity of literature examining risk factors for ARDS in septic patients early in their course. This study examined the role of early fluid administration and identified other risk factors within the first 6 h of hospital presentation associated with developing ARDS in septic patients.

**Methods:**

This was a secondary analysis of septic adult patients presenting to the Emergency Department or being admitted for high-risk elective surgery from the multicenter observational cohort study, US Critical Injury and Illness trial Group-Lung Injury Prevention Study 1 (USCIITG-LIPS 1, NCT00889772). Multivariable logistic regression was performed to identify potential early risk factors for ARDS. Stratified analysis by shock status was performed to examine the association between early fluid administration and ARDS.

**Results:**

Of the 5584 patients in the original study cohort, 2534 (45.4%) met our criteria for sepsis. One hundred and fifty-six (6.2%) of these patients developed ARDS during the hospital stay. In multivariable analyses, Acute Physiology and Chronic Health Evaluation (APACHE) II score (OR 1.10, 95% CI 1.07–1.13), age (OR 0.97, 95% CI 0.96–0.98), total fluid infused in the first 6 h (in liters) (OR 1.15, 95% CI 1.03–1.29), shock (OR 2.57, 95% CI 1.62–4.08), pneumonia as a site of infection (OR 2.31, 95% CI 1.59–3.36), pancreatitis (OR 3.86, 95% CI 1.33–11.24), and acute abdomen (OR 3.77, 95% CI 1.37–10.41) were associated with developing ARDS. In the stratified analysis, total fluid infused in the first 6 h (in liters) (OR 1.05, 95% CI 0.87–1.28) was not associated with the development of ARDS in the shock group, while there was an association in the non-shock group (OR 1.21, 95% CI 1.05–1.38).

**Conclusions:**

In septic patients, the following risk factors identified within the first 6 h of hospital presentation were associated with ARDS: APACHE II score, presence of shock, pulmonary source of infection, pancreatitis, and presence of an acute abdomen. In septic patients without shock, the amount of fluid infused during the first 6 h of hospital presentation was associated with developing ARDS.

**Electronic supplementary material:**

The online version of this article (doi:10.1186/s13613-017-0233-1) contains supplementary material, which is available to authorized users.

## Background

Acute respiratory distress syndrome (ARDS) is a common condition encountered in the intensive care unit (ICU), with close to 10% of all patients admitted to the ICU developing ARDS [1]. Sepsis has long been recognized as a major risk factor for the development of ARDS. Prior investigations have reported approximately up to 40% of ARDS patients also having a diagnosis of sepsis [2, 3]. Previous work has described risk factors in septic shock patients that are predictive of ARDS, but this work has largely focused on patients admitted to the ICU [4]. Recent international sepsis guidelines have highlighted the importance of early recognition and treatment and have specifically focused on the first 3 and 6 h of care [5]. Multiple studies have demonstrated improved mortality and outcomes with increased adherence to these guidelines [6, 7]. It is likely that during this critical 6-h period of initial presentation there are readily identifiable risk factors in the sepsis population that predispose them to developing ARDS. Despite these observations, literature examining risk factors for ARDS in septic patients early in their course like in the emergency department remains sparse. There have been preliminary data on the epidemiology of ARDS in septic patients in the emergency department, but these studies have been limited by their retrospective nature, only including a single center, and small sample size [8, 9].

Early fluid administration may be an important modifiable risk factor for the development of ARDS in sepsis patients. There has been recent debate regarding the optimal fluid strategy in septic patients. One of the most important components of sepsis resuscitation bundles is fluid resuscitation. Three recently published sepsis trials found that protocolized resuscitation did not perform any better than usual or standard care by physicians [10–12]. The mortality rates in these studies were much lower than prior studies, and this has led to speculation that the early aggressive volume resuscitation instituted in these studies partially explains this observed lower mortality. On the other hand, there have been several studies demonstrating worse outcomes with larger fluid resuscitation and positive fluid balance during ICU stay in septic patients [13–16]. Sepsis is a highly inflamed state, with increased capillary permeability. Excessive volume administration could lead to increased pulmonary edema and subsequent ARDS. In spite of this, the role of volume resuscitation and developing ARDS during the early period of sepsis has not been extensively studied.

In a large multicenter cohort of septic patients, we sought to identify risk factors readily detectable during the first 6 h of hospital presentation that were associated with the development of ARDS and examine the association of fluid administration during the first 6 h and ARDS.

## Methods

### Study design and setting

This was a secondary analysis of a multicenter observational cohort study, US Critical Injury and Illness trial Group-Lung Injury Prevention Study 1 (USCIITG-LIPS 1, NCT00889772) [17]; patients were enrolled prospectively in 19 hospitals and retrospectively (after hospital discharge) in three hospitals over a 6-month period, beginning in March 2009. The hospitals included both community and academic medical centers with 20 of the hospitals located in the USA and two hospitals located in Turkey. The study was approved by the institutional review board at each participating institution. Approval was also granted for ancillary studies such as the present investigation.

### Study patients

The original study included consecutive adult patients with one or more study-defined ARDS risk factors admitted to the hospital through the Emergency Department or admitted for high-risk elective surgery. This was a subgroup analysis that included patients with sepsis as an ARDS risk factor. These patients were followed during their initial hospital stay to assess for the development of ARDS. We defined sepsis as those with the presence of known or suspected infection with 2 or more systemic inflammatory response syndrome (SIRS) criteria or the diagnosis of pneumonia at the time of enrollment. Patients with the diagnosis of ARDS at the time of initial presentation were excluded.

### Data collection

As detailed in the original lung injury prediction score (LIPS) study [17], demographics, comorbidities, and clinical variables were collected during the first 6 h of initial evaluation. Data were entered into a secure electronic database (REDCap).

### Outcome

The primary outcome was development of ARDS during the hospital stay. ARDS was defined according to the Berlin definition [18]. The Berlin definition was retrospectively applied to the data, as this definition was not yet published at the time of the data collection.

### Statistical analysis

Continuous data were reported as means and standard deviations. Categorical data were reported as counts and percentages. As appropriate, Student’s *t* tests and Chi-square tests were used to compare characteristics between the ARDS and non-ARDS groups. Logistic regression was performed to examine the association of potential risk factors and development of ARDS in this sepsis cohort. We a priori hypothesized the following risk factors would be associated with ARDS in septic patients: Acute Physiology and Chronic Health Evaluation (APACHE) II score, age, total fluid infused during first 6 h, presence of shock, race, gender, pneumonia as site of infection, and blood product transfusion. Shock was defined as presence of hypotension (systolic blood pressure <90 mmHg, or decrease of 40 mmHg from baseline, or mean arterial pressure <65 mmHg) with evidence of inadequate tissue perfusion on physical examination (altered mental status not explained by other causes other than the hemodynamic status and urine output less than 0.5 ml/Kg/min). The definitions of all clinical variables are available in the online data supplement of the original study [17]. Total fluid infused during first 6 h was calculated by adding the total amount of crystalloid, colloid, and other infusions received during that time period. We additionally examined the risk factors identified in the original LIPS study that were associated with ARDS [17].

We performed univariable analysis of the risk factors identified in the original LIPS study. Risk factors with a *p* value <0.2 were then entered into a multivariable model. Additionally, using a forced entry strategy, the a priori hypothesized risk factors were also entered into the multivariable model. We then used stepwise backward elimination, retaining variables with a *p* value <0.2, to select the optimal model. Variables in which less than 1% of the study population had the variable present, or in which >30% of the values were missing were excluded from analysis. We additionally hypothesized that the association between total amount of fluid infused during the first 6 h and development of ARDS would differ between the shock and non-shock groups and thus performed a stratified analysis by shock status.

Odds ratio (OR) and 95% CI were reported. Imputation with mean value was used to analyze continuous variables with missing data. Missing indicator method was used to analyze categorical variables with missing data. The following variables had >5% missing data: total fluid infused during first 6 h (28%), alcohol use (10%), blood product transfusion (23%), obesity (20%), tobacco use (7%), and FIO_2_ >0.35 (8%). Sensitivity analysis was performed using complete case analysis. All analyses were performed using SAS version 9.4 (SAS Institute, Cary, NC). In the final model, a *p* value <0.05 was considered significant.

## Results

**Fig. 1:**
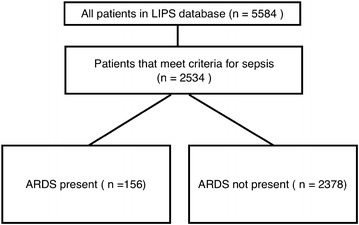
Patient selection diagram with outcomes

A total of 5584 patients were included in the original LIPS study cohort (309 of these patients were enrolled retrospectively). Out of 5584, 2534 patients (45.4%) in the original study cohort met our predefined criteria for sepsis and were analyzed (Fig. 1). One hundred and fifty-six (6.2%) of these patients developed ARDS during the hospital stay. Mean time to development of ARDS was 4.5 ± 5.3 days, with approximately 50% of the cases occurring in the first 2 days of hospitalization, and 80% occurring within 5 days. 1209 (47.7%) of the sepsis cohort were admitted to the ICU, and 170 (6.7%) died during their in-hospital stay. Of the patients that developed ARDS, 54 (34.6%) died, while 116 (4.9%) of the patients that did not develop ARDS died. The mean hospital length of stay for septic patients with ARDS was 19.1 ± 16.2 days and for those without ARDS was 7.6 ± 8.1 days (*p* < 0.001). Patient characteristics are listed in Table 1.

**Table 1:** Characteristics of patients in the sepsis cohort

	Total (*n* = 2534)	ARDS (*n* = 156)	No ARDS (*n* = 2378)	*p* value
APACHE II	11.7 ± 6.4	16.3 ± 7.4	11.4 ± 6.2	<0.001
Age (years)	58.5 ± 18.8	54.9 ± 17.3	58.8 ± 18.9	0.01
Total fluid infused during first 6 h (L)	1.49 ± 1.51	2.54 ± 2.31	1.41 ± 1.42	<0.001
Race				0.04
White	1448 (59.2)	97 (63.8)	1351 (58.9)	
Black	724 (29.6)	31 (20.4)	693 (30.2)	
Asian	35 (1.4)	3 (2.0)	32 (1.4)	
Other	240 (9.8)	21 (13.8)	219 (9.5)	
Shock	234 (9.23)	44 (28.2)	190 (8.0)	<0.001
Male	1298 (51.2)	94 (60.3)	1204 (50.6)	0.02
Pneumonia as site of infection	1234 (48.7)	91 (58.33)	1143 (48.1)	0.01
Alcohol use	596 (26.0)	41 (29.3)	555 (25.8)	0.36
Blood product transfusion	51 (2.6)	10 (8.6)	41 (2.2)	<0.001
Aspiration	103 (4.1)	15 (9.6)	88 (3.7)	<0.001
Pancreatitis	24 (1.0)	5 (3.2)	19 (0.8)	0.003
Thoracic surgery	6 (0.2)	0 (0.0)	6 (0.3)	0.53
Spine surgery	3 (0.1)	2 (1.3)	1 (0.0)	<0.001
Acute abdomen	42 (1.7)	6 (3.9)	36 (1.5)	0.03
Cardiac surgery	3 (0.1)	0 (0.0)	3 (0.1)	0.66
Aortic surgery	3 (0.1)	0 (0.0)	3 (0.1)	0.66
Lung contusion	2 (0.1)	0 (0.0)	2 (0.1)	0.72
Near drowning	0 (0)	0	0	NA
Brain injury	11 (0.4)	3 (1.9)	8 (0.34)	0.004
Smoke inhalation	4 (0.2)	1 (0.6)	3 (0.1)	0.12
Long-bone fractures	5 (0.2)	3 (1.9)	2 (0.1)	<0.001
Obesity (BMI > 30)	598 (23.6)	44 (28.2)	554 (23.3)	0.16
Chemotherapy	131 (5.2)	10 (6.4)	121 (5.1)	0.47
Diabetes mellitus	733 (28.9)	33 (21.1)	700 (29.4)	0.03
Tobacco use				0.83
Never	1177 (49.7)	73 (51.4)	1104 (49.6)	
Former	652 (27.6)	36 (25.4)	616 (27.7)	
Current	538 (22.7)	33 (23.3)	505 (22.7)	
Emergency surgery	41 (1.6)	5 (3.2)	36 (1.5)	0.10
Tachypnea (RR > 30)	239 (9.7)	30 (20.0)	209 (9.0)	<0.001
Hypoxemia (SpO_2_ < 95%)	861 (34.6)	68 (44.2)	793 (34.0)	0.01
FIO_2_ >0.35	395 (17.0)	61 (41.2)	334 (15.4)	<0.001
Hypoalbuminemia	695 (54.9)	64 (74.4)	631 (53.4)	<0.001
Acidosis (pH < 7.35)	231 (42.5)	50 (55.6)	181 (40.0)	0.006

Data are presented as mean ± SD or *n* (%) unless otherwise indicated

*APACHE* Acute Physiology and Chronic Health Evaluation, *BMI* body mass index, *RR* respiratory rate

In univariable analysis, APACHE II score, age, total fluid infused during first 6 h, shock, race, gender, pneumonia, blood product transfusion, aspiration, pancreatitis, acute abdomen, tachypnea, hypoxemia, and FIO_2_ >0.35 were all associated with the increased odds of development of ARDS, while the diagnosis of diabetes mellitus was found to be protective against ARDS (Table 2).

**Table 2:** Univariable logistic regression of early risk factors for ARDS in sepsis cohort

	Odds ratio (95% CI)	*p* value
APACHE II	1.11 (1.08–1.13)	<0.001
Age (years)	0.99 (0.98–1.0)	0.01
Total fluid infused during first 6 h (L)	1.40 (1.28–1.54)	<0.001
Race		
White	Reference	Reference
Black	0.62 (0.41–0.94)	0.02
Asian	1.31 (0.39–4.34)	0.59
Other	1.34 (0.82–2.19)	0.25
Shock	4.52 (3.10–6.61)	<0.001
Gender (male)	1.48 (1.06–2.06)	0.02
Pneumonia as site of infection	1.51 (1.09–2.10)	0.01
Alcohol use	1.19 (0.82–1.74)	0.36
Blood product transfusion	4.10 (2.00–8.41)	<0.001
Aspiration	2.77 (1.56–4.91)	<0.001
Pancreatitis	4.11 (1.51–11.16)	0.003
Acute abdomen	2.60 (1.08–6.27)	0.03
Obesity (BMI > 30)	1.29 (0.90–1.86)	0.16
Chemotherapy	1.28 (0.66–2.49)	0.47
Diabetes mellitus	0.64 (0.43–0.95)	0.03
Tobacco use		
Never	Reference	Reference
Former	0.88 (0.59–1.33)	0.56
Current	0.99 (0.65–1.51)	0.81
Emergency surgery	2.16 (0.83–5.57)	0.11
Tachypnea (RR > 30)	2.52 (1.65–3.86)	<0.001
Hypoxemia (SpO_2_ < 95%)	1.54 (1.11–2.14)	0.01
FIO_2_ >0.35	3.86 (2.72–5.46)	<0.001

For continuous variables, the odds ratio indicates the increased odds of ARDS for a 1-unit increase of the variable

*APACHE* Acute Physiology and Chronic Health Evaluation, *BMI* body mass index, *RR* respiratory rate

The final multivariable model is demonstrated in Table 3. APACHE II score (OR 1.10, 95% CI 1.07–1.13), age (OR 0.97, 95% CI 0.96–0.98), total fluid infused in the first 6 h (in liters) (OR 1.15, 95% CI 1.03–1.29), shock (OR 2.57, 95% CI 1.62–4.08), pneumonia as a site of infection (OR 2.31, 95% CI 1.59–3.36), pancreatitis (OR 3.86, 95% CI 1.33–11.24), and acute abdomen (OR 3.77, 95% CI 1.37–10.41) were all associated with the development of ARDS. We performed a sensitivity analysis with complete case analysis that yielded similar results (Additional file 1: Table S1).

**Table 3:** Multivariable logistic regression of early risk factors for ARDS in sepsis cohort

	Odds ratio (95% CI)	*p* value
APACHE II	1.10 (1.07–1.13)	<0.001
Age (years)	0.97 (0.96–0.98)	<0.001
Total fluid infused during first 6 h (L)	1.15 (1.03–1.29)	0.01
Shock	2.57 (1.62–4.08)	<0.001
Gender (male)	1.30 (0.92–1.85)	0.14
Race		
White	Reference	Reference
Black	0.56 (0.36–0.87)	0.08
Asian	1.14 (0.32–4.11)	0.58
Other	1.14 (0.66–1.96)	0.29
Pneumonia as site of infection	2.31 (1.59–3.36)	<0.001
Pancreatitis	3.86 (1.33–11.24)	0.01
Acute abdomen	3.77 (1.37–10.41)	0.01
Diabetes mellitus	0.74 (0.48–1.12)	0.16
Tachypnea (RR > 30)	1.41 (1.00–1.97)	0.05

For continuous variables, the odds ratio indicates the increased odds of ARDS for a 1-unit increase in the variable

*APACHE* Acute Physiology and Chronic Health Evaluation, *RR* respiratory rate

**Fig. 2:**
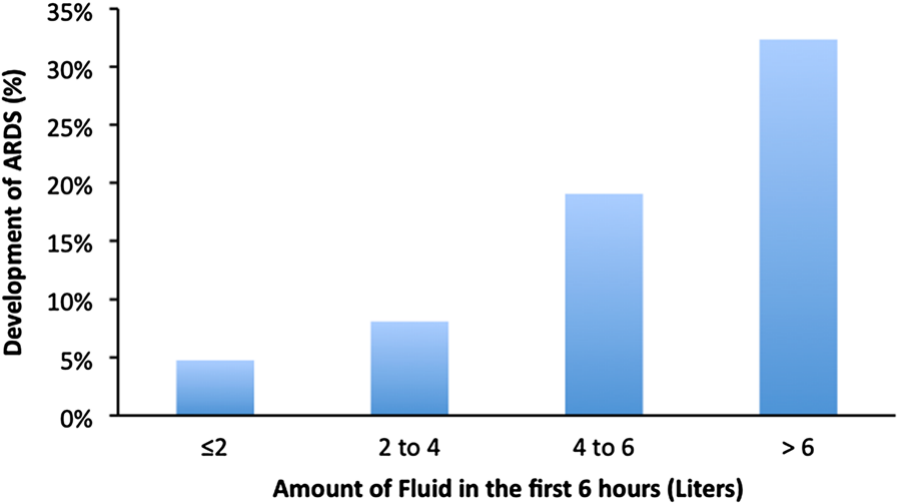
Frequency of acute respiratory distress syndrome (ARDS) development according to amount of fluid administered during the first 6 h of hospital presentation

We also observed that during the first 6 h of hospital presentation the incidence of ARDS increased with increasing fluid administration (Fig. 2). The stratified analysis according to the presence of shock revealed that the relationship between amount of fluid infused in first 6 h and the development of ARDS was not present within the subgroup of patients with shock (OR 1.05, 95% CI 0.87–1.28) (Table 4). This association was still present in the non-shock group (OR 1.21, 95% CI 1.05–1.38).

**Table 4:** Shock subgroup analysis: multivariable analysis of total volume in first 6 h and the development of ARDS

	Odds ratio (95% CI)	*p* value
Shock	1.05 (0.87–1.28)	0.60
No shock	1.21 (1.05–1.38)	0.01

The odds ratio indicates the increased odds of ARDS for a 1-l increase in volume of fluids administered

## Discussion

In this large cohort of patients with sepsis and pneumonia, we found that the rate of developing ARDS was low. Although only 6% of at-risk patients developed ARDS, mortality was significantly higher in those who developed ARDS. We found that APACHE II score, age, higher volume of early fluid administration, pulmonary source of sepsis, shock, pancreatitis, and acute abdomen were all associated with the development of ARDS. Of these exposures, fluid administration appears to be the only potentially modifiable exposure.

Our results highlight the role of the amount of fluid administered to septic patients during the first 6 h of care and the development of ARDS. Other studies support our findings that increased fluid administration may be associated with the development of ARDS. Jia et al. [19] demonstrated that a positive fluid balance during the first 48 h of mechanically ventilated patients is associated with the development of ARDS. In addition, after initial resuscitation, conservative fluid-management strategies compared to liberal strategies have increased days alive and off the ventilator in patients with established ARDS, many of whom had sepsis as a risk factor [20]. Multiple studies have demonstrated that increasing extravascular lung water is associated with mortality in ARDS patients [21–23]. A positive fluid balance has also been associated with increased mortality in septic shock patients [13].

Conversely, early resuscitation in sepsis has been shown to decrease inflammatory markers [24]. Therefore, fluid resuscitation during this early phase of sepsis may actually be beneficial and decrease the risk of ARDS by limiting the inflammatory cascade. One study demonstrated in septic patients with the diagnosis of ARDS aggressive fluid resuscitation in the first 6 h followed by conservative fluid management in the next 7 days was the optimal fluid therapy in terms of mortality [25]. A prior investigation demonstrated that inadequate resuscitation for patients in septic shock was an independent risk factor for ARDS [4]. Another study demonstrated that total volume of fluid infused during the first 24 h of care of severe sepsis and septic shock patients did not increase risk of ARDS [26].

Our stratified analysis showed that fluid administration during the first 6 h was not significantly associated with ARDS in the subgroup of patients with shock. However, we did demonstrate that the association between amount of fluid infused in the first 6 h and the development of ARDS was present in patients without shock. This may suggest that septic patients without shock are at the highest risk of ARDS with early excessive fluid administration. It is important to note that only 9% of our patients had shock. Larger size studies with sufficient power are necessary to examine this issue. Interestingly, this phenomenon has also been observed in patients with severe blunt trauma. A multicenter prospective study demonstrated that blunt trauma patients with prehospital hypotension and higher crystalloid infusion did not have an increased incidence of ARDS, while blunt trauma patients without prehospital hypotension and higher crystalloid infusion did have a higher incidence of ARDS [27].

Despite multiple randomized control trials comparing different early resuscitation protocols for septic patients, this relationship between early fluid resuscitation in septic patients and ARDS has not been well studied. Unfortunately, none of these trials specifically looked at ARDS as an outcome. The original early goal-directed therapy (EGDT) trial did not measure the incidence of ARDS directly, but did report the need for mechanical ventilation [28]. The patients randomized to the EGDT group received more fluids in the first 6 h and had a significantly decreased requirement of mechanical ventilation. This suggests in that patient population early aggressive fluid administration was not associated with respiratory impairment and in fact was associated with improved respiratory outcomes. Our results do not contradict those findings. The patients in the EGDT study were considerably sicker than our cohort. They had an initial APACHE score of 21 and an overall mortality of 37%. As we have demonstrated in our study, it is in the less sick patients without shock in which we observed the association of increased early fluids and ARDS. The subsequent three sepsis trials comparing standard care to EGDT did not demonstrate a significant association between EGDT and respiratory outcomes [10–12]. This may be due to the fact that there was not a large difference in amount of fluid received between the control and interventional arms. In the PROMISE and ARISE trials, the difference in the amount of fluids given in the first 6 h between the control and intervention groups was between 200 and 250 cc. However, in the PROCESS trial there were three arms: EGDT, another protocolized resuscitation, and standard care. The standard care group had a significantly lower volume infused in the first 6 h: 2.3L (standard care) versus 2.8L (EGDT) and 3.3L (other protocolized resuscitation) (*p* < 0.001) and had a trend toward less respiratory failure. At this point, it is unclear what the optimal fluid strategy is during the early phase of sepsis to prevent ARDS. It likely depends on several factors including severity of illness and hemodynamic status.

Our findings are also consistent with prior investigations that demonstrated severity of illness, pneumonia as a source of infection, and shock at presentation as risk factors for ARDS in septic patients [8]. We additionally found that pancreatitis and acute abdomen were risk factors for ARDS in this cohort. This is not surprising, given that lung and abdomen have been identified as the most frequent sources of infection in patients with ARDS [29]. The contribution of pulmonary sepsis to ARDS is likely multifactorial. Septic patients with pneumonia have a direct lung injury from the pneumonia itself and then indirect lung injury from the sepsis inflammatory cascade, which can both lead to ARDS. It has also long been known that ARDS is a major complication of severe pancreatitis. ARDS has been reported to be the major cause of death of acute pancreatitis patients that die within one week of presentation [30, 31].

Blood product administration is a known risk factor for lung injury and progression to ARDS in critically ill patients [19, 32–34]. Our study found an association with ARDS in univariable analysis, but our multivariable analysis did not. We were likely underpowered to demonstrate such an association, since only 2.6% of our patients received blood products. In contrast to our results, Iscimen et al. [4] found that blood product transfusion in the septic shock population independently predicted ARDS. Their study differed from ours in that it only evaluated patients in septic shock, and over 50% of the patients received some blood product transfusion.

Our study has several strengths. This was a large multicenter study, and the majority of data were prospectively collected. Additionally, our study is generalizable to patients with the entire spectrum of sepsis, since our study included sepsis and septic shock. Our study also has some limitations. First, we did not collect data on some important risk factors that have been associated with ARDS in sepsis including time to antibiotics and lactate level. Second, we have incomplete data on some of the covariates. We dealt with the missing data using established epidemiologic methods. We also performed a sensitivity analysis with complete case analysis and found that our results were similar. Third, there is risk for misclassification from medical chart review. This risk was reduced since the vast majority of these patients were enrolled prospectively with close follow-up. Fourth, there is the limitation that most ARDS investigations share regarding the reproducibility of diagnosis of ARDS. In order to mitigate this limitation, mandatory structured training in ARDS assessment was instituted and site-principal investigators were responsible for ensuring quality. Finally, we are able to demonstrate association, but not demonstrate causality. Future studies, including advanced adjustment techniques, are warranted to confirm the relationship between initial fluid administration and ARDS development. However, because the application of propensity score methods for continuous exposures is less well developed than their use for binary exposures, we adjusted for potential confounders using multivariable logistic regression models [35].

## Conclusions

In septic patients, we demonstrated that the following variables present upon initial hospital presentation: Severity of illness, age, pulmonary source of sepsis, presence of shock, pancreatitis, and acute abdomen were associated with developing ARDS. In septic patients without shock, we also identified another important association between a potentially modifiable risk factor, the amount of fluid infused in the first 6 h, and the development of ARDS. Future investigations should focus on determining the optimal early resuscitation strategies for septic patients based on severity of sepsis and examine the outcome of ARDS.

## Appendix: US Critical Illness and Injury Trials Group: Lung Injury Prevention Study Participating Centers and Investigators

Mayo Clinic, Rochester, Minnesota: Adil Ahmed, M.D.; Ognjen Gajic, M.D.; Michael Malinchoc, M.S.; Daryl J. Kor, M.D.; Bekele Afessa, M.D.; Rodrigo Cartin-Ceba, M.D.; Rickey E. Carter, Ph.D.; Departments of Internal Medicine, Health Sciences Research and Anesthesiology

University of Missouri, Missouri, Columbia: University of Missouri-Columbia: Ousama Dabbagh, M.D., M.S.P.H., Associate Professor of Clinical Medicine; Nivedita Nagam, M.D.; Shilpa Patel, M.D.; Ammar Kar; and Brian Hess

University of Michigan, Ann Arbor, Michigan: Pauline K. Park, M.D., F.A.C.S., F.C.C.S., Co-Director, Surgical Intensive Care Unit, Associate Professor, Surgery; Julie Harris, Clinical Research Coordinator; Lena Napolitano, M.D.; Krishnan Raghavendran, M.B.B.S.; Robert C. Hyzy, M.D.; James Blum, M.D.; Christy Dean

University of Texas Southwestern Medical Center in Dallas, Dallas, Texas: Adebola Adesanya, M.D.; Srikanth Hosur, M.D.; Victor Enoh, M.D.; Department of Anesthesiology, Division of Critical Care Medicine

University of Medicine and Dentistry of New Jersey, New Jersey: Steven Y. Chang, Ph.D., M.D., Assistant Professor, MICU Director, Pulmonary and Critical Care Medicine; Amee Patrawalla, M.D., M.P.H.; Marie Elie, M.D.

Brigham and Women’s Hospital, Boston, Massachusetts: Peter C. Hou, M.D.; Jonathan M. Barry, B.A.; Ian Shempp, B.S.; Atul Malhotra, M.D.; Gyorgy Frendl, M.D., Ph.D.; Departments of Emergency Medicine, Surgery, Internal Medicine and Anesthesiology,

Perioperative and Pain Medicine, Division of Burn, Trauma, and Surgical Critical Care

Wright State University Boonshoft School of Medicine and Miami Valley Hospital, Dayton, Ohio: Harry Anderson III, M.D., Professor of Surgery; Kathryn Tchorz, M.D., Associate Professor of Surgery; Mary C. McCarthy, M.D., Professor of Surgery; David Uddin, Ph.D., D.A.B.C.C., C.I.P., Director of Research

Wake Forest University Health Sciences, Winston-Salem, North Carolina: J. Jason Hoth, M.D., Assistant Professor of Surgery; Barbara Yoza, Ph.D., Study Coordinator

University of Pennsylvania, Philadelphia, Pennsylvania: Mark Mikkelsen, M.D., M.S.C.E., Assistant Professor of Medicine, Pulmonary, Allergy, and Critical Care Division; Jason D. Christie, M.D.; David F. Gaieski, M.D.; Paul Lanken, M.D.; Nuala Meyer, M.D.; Chirag Shah, M.D.

Temple University School of Medicine, Philadelphia, Pennsylvania: Nina T. Gentile, M.D., Associate Professor and Director, Clinical Research, Department of Emergency Medicine, Temple University School of Medicine; Karen Stevenson, M.D., Resident, Department of Emergency Medicine; Brent Freeman, B.S., Research Coordinator; Sujatha Srinivasan, M.D., Resident, Department of Emergency Medicine

Mount Sinai School of Medicine, New York, New York: Michelle Ng Gong, M.D., M.S., Assistant Professor, Pulmonary, Critical Care, and Sleep Medicine, Department of Medicine

Beth Israel Deaconess Medical Center, Boston, Massachusetts: Daniel Talmor, M.D., Director of Anesthesia and Critical Care, Associate Professor of Anaesthesia, Harvard Medical School, Boston, Massachusetts; S. Patrick Bender, M.D.; Mauricio Garcia, M.D.

Massachusetts General Hospital Harvard Medical School, Boston, Massachusetts: Ednan Bajwa, M.D., M.P.H., Instructor in Medicine; Atul Malhotra, M.D., Assistant Professor; B. Taylor Thompson, Associate Professor; David C. Christiani, M.D., M.P.H., Professor

University of Washington, Harborview, Seattle, Washington: Timothy R. Watkins, M.D., Acting Instructor, Department of Medicine, Division of Pulmonary and Critical Care Medicine; Steven Deem, M.D.; Miriam Treggiari, M.D., M.P.H.

Mayo Clinic Jacksonville: Emir Festic, M.D.; Augustine Lee, M.D.; John Daniels, M.D.

Akdeniz University, Antalyia, Turkey: Melike Cengiz, M.D., Ph.D.; Murat Yilmaz, M.D.

Uludag University, Bursa, Turkey: Remzi Iscimen, M.D.

Bridgeport Hospital, Yale New Haven Health, New Haven, Connecticut: David Kaufman, M.D., Section Chief, Pulmonary, Critical Care, and Sleep Medicine, Medical Director, Respiratory Therapy

Emory University, Atlanta, Georgia: Annette Esper, M.D.; Greg Martin, M.D.

University of Illinois at Chicago, Chicago, Illinois: Ruxana Sadikot, M.D., M.R.C.P.

University of Colorado, Boulder, Colorado: Ivor Douglas, M.D.

Johns Hopkins University: Jonathan Sevransky, M.D., M.H.S., Assistant Professor of Medicine, Medical Director, JHBMC MICU.

## Additional file

**Additional file 1: Table S1.** Sensitivity analysis using complete case analysis (1603 total patients included). Multivariable logistic regression of early risk factors for ARDS in sepsis cohort.

## Abbreviations

ARDS: acute respiratory distress syndrome
APACHE: Acute Physiology and Chronic Health Evaluation
ICU: intensive care unit
LIPS: lung injury prediction score
OR: odds ratio
SIRS: systemic inflammatory response syndrome
