# Supplementary material for: Early risk factors and the role of fluid administration in developing acute respiratory distress syndrome in septic patients
Source: Ann Intensive Care. 2017 Jan 23;7:11. doi: 10.1186/s13613-017-0233-1 (PMC5256622; doi:10.1186/s13613-017-0233-1)
Supplement: Supplementary file 1 — Additional file 1: Table S1. Sensitivity analysis using complete case analysis (1603 total patients included). Multivariable logistic regression of early risk factors for ARDS in sepsis cohort. [file 13613_2017_233_MOESM1_ESM.docx]

Table S1: Sensitivity analysis using complete case analysis (1603 total patients included). Multivariable logistic regression of early risk factors for ARDS in sepsis cohort.

|  | **Odds ratio (95% CI)** | **p-value** |
| --- | --- | --- |
| **APACHE II** | 1.10 (1.06-1.14) | <0.001 |
| **Age (years)** | 0.97 (0.95 - 0.98) | <0.001 |
| **Total fluid infused during first 6 hours (L)** | 1.16 (1.02-1.31) | 0.02 |
| **Shock** | 2.35 (1.28-4.30) | 0.006 |
| **Gender (male)** | 1.27 (0.82-1.97) | 0.29 |
| **Race** |  |  |
| **White** | Reference | Reference |
| **Black** | 0.49 (0.28-0.85) | 0.11 |
| **Asian** | 0.89 (0.11-7.44) | 0.94 |
| **Other** | 1.11 (0.56-2.18) | 0.44 |
| **Pneumonia as site of infection** | 2.30 (1.41-3.73) | 0.001 |
| **Pancreatitis** | 20.84 (4.88-89.12) | <0.001 |
| **Acute abdomen** | 11.71 (3.19-43.07) | <0.001 |
| **Diabetes mellitus** | 0.98 (0.59-1.63) | 0.94 |
| **Tachypnea (RR > 30)** | 1.97 (1.14 -3.39) | 0.01 |

APACHE = Acute Physiology and Chronic Health Evaluation, RR = respiratory rate.

For continuous variables, the odds ratio indicates the increased odds of ARDS for a 1-unit increase of the variable.
